# FLIM data analysis based on Laguerre polynomial decomposition and machine-learning

**DOI:** 10.1117/1.JBO.26.2.022909

**Published:** 2021-01-07

**Authors:** Shuxia Guo, Anja Silge, Hyeonsoo Bae, Tatiana Tolstik, Tobias Meyer, Georg Matziolis, Michael Schmitt, Jürgen Popp, Thomas Bocklitz

**Affiliations:** a University of Jena, Institute of Physical Chemistry, Abbe Center of Photonics, Jena, Germany; b Leibniz Institute of Photonic Technology, Member of Leibniz Health Technologies, Jena, Germany; c Jena University Hospital, Jena, Germany

**Keywords:** machine learning, fluorescence lifetime imaging microscopy, life time extraction, chemometrics, fit-free

## Abstract

**Significance**: The potential of fluorescence lifetime imaging microscopy (FLIM) is recently being recognized, especially in biological studies. However, FLIM does not directly measure the lifetimes, rather it records the fluorescence decay traces. The lifetimes and/or abundances have to be estimated from these traces during the phase of data processing. To precisely estimate these parameters is challenging and requires a well-designed computer program. Conventionally employed methods, which are based on curve fitting, are computationally expensive and limited in performance especially for highly noisy FLIM data. The graphical analysis, while free of fit, requires calibration samples for a quantitative analysis.

**Aim**: We propose to extract the lifetimes and abundances directly from the decay traces through machine learning (ML).

**Approach**: The ML-based approach was verified with simulated testing data in which the lifetimes and abundances were known exactly. Thereafter, we compared its performance with the commercial software SPCImage based on datasets measured from biological samples on a time-correlated single photon counting system. We reconstructed the decay traces using the lifetime and abundance values estimated by ML and SPCImage methods and utilized the root-mean-squared-error (RMSE) as marker.

**Results**: The RMSE, which represents the difference between the reconstructed and measured decay traces, was observed to be lower for ML than for SPCImage. In addition, we could demonstrate with a three-component analysis the high potential and flexibility of the ML method to deal with more than two lifetime components.

**Conclusions**: The ML-based approach shows great performance in FLIM data analysis.

## 1 Introduction

Fluorescence is one of the relaxation pathways after a fluorophore is promoted by a photon to its electric excited state. The resulting fluorescence emission can be characterized by its intensity (I), frequency (ω), and lifetime (τ). Out of these parameters, the lifetime τ is independent of the measurement parameters, such as the excitation intensity and fluorophore concentrations. It is also unaffected by photobleaching. However, the variations in the microenvironment, such as the pH values, temperature, polarity, and the presence of quenchers, are well reflected in the lifetime changes.^1^^,^^2^ Thus, the fluorescence lifetime can provide new insights into samples that cannot be characterized by its fluorescence intensity because of overlapping florescence spectra. These facts make fluorescence lifetime imaging microscopy (FLIM) increasingly popular as a powerful complement to fluorescence intensity imaging and is believed useful especially for the study of dynamic biological processes. FLIM in combination with Förster resonance energy transfer (FRET) enabled the investigation of protein–protein interactions, biosensor activities, and ligand-receptor engagements.^3^ The potential of FLIM was reflected in studying the metabolic activities,^4^ the drug delivery and release,^5^ the basic mechanisms of diseases,^6^ and medical diagnostics.^7^^–^^9^

In practice, the measurement of fluorescence lifetime can be achieved in frequency or time domain.^10^^–^^12^ The frequency-domain FLIM uses modulated sinusoidal or pulsed excitation and measures the changes of the modulation depth and phase of the fluorescence emission with respect to the excitation signal. Measurement in time-domain is conducted by either time-correlated single photon counting (TCSPC) or time-gated detection. The two techniques are based on laser scanning microscopes or white light microscopes, respectively. Systems of the former case record the arrival time of each single photon and eventually construct a histogram of the photon counts over time bins. In the latter case, the photons are collected periodically after the excitation, forming a fluorescence decay over time. In this paper, we will focus exclusively on the TCSPC measurements as it was used in our experiments. Nonetheless, the two time-domain measurements put no fundamental difference in the subsequent data analysis, and we can refer by “decay trace” to both of the histogram and the fluorescence decay without discrimination.

The output of an FLIM system is normally a three-dimensional (3-D) data cube, with x- and y-axes as the spatial coordinates and the t-axis as the time coordinate. That is to say, a decay trace is acquired at each of the measurement positions (x,y), which is represented as Eq. (1). This model is composed of exponential decay traces superposed from all lifetime components in the sample, and the result is convolved by the instrumental response function (IRF) R in the time domain:

$$I(t)=\int_{0}^{t}R(t-T)\underset{i}{\sum }\alpha_{i}\;\exp(-\frac{T}{\tau_{i}})dT.$$ (1)

The essence of applying FLIM, regardless of the measurement mechanisms, is to extract the lifetime information $\tau_{i}$ and their abundances $\alpha_{i}$. The results of $\tau_{i}$ are essential for identifying molecular species within the samples, whereas the abundances $\alpha_{i}$ represent the concentration of the molecular species. In this context, it is required to precisely estimate the lifetimes $\tau_{i}$ and abundances $\alpha_{i}$ from the measured decay traces. The straightforward approach is curve fitting,^13^^–^^15^ in which $\tau_{i}$ and $\alpha_{i}$ are optimized so the reconstruction from Eq. (1) matches the measured decay trace. This method is often based on algorithms such as maximum likelihood, least squares fit, or Bayesian analysis. The fit can be conducted in either an individual or global manner. The individual fit calculates the lifetimes $\tau_{i}$ and abundances $\alpha_{i}$ for each decay trace separately, which largely preserves the spatial characteristics of the sample. However, the fit procedure is computationally intensive. Approaches such as variable projection can speed up the calculation but are still time-consuming.^16^ An adequately fast curve fit requires advanced technologies such as parallel computation or optimizations using GPUs (graphic processing units), which adds to the cost of the device significantly. In addition, the fit is sensitive to noise and it is extremely challenging to fit data with low photon-counts. Preprocessing steps, such as denoising^17^ or photon count upgrading,^18^ may help to improve the fits, but these methods can introduce a bias as well. Alternatively, it is possible to assume that the lifetimes are homogeneous in the sample so the lifetimes can be optimized based on all decay traces together, and only the abundances are calculated individually. This represents the idea of a global fit.^15^ It is faster and more robust to noise. However, this technique applies only if the lifetimes do not change spatially and are inadequate for heterogeneous samples. In this context, the stretched-exponential function was shown to produce better results,^19^ which was yet limited to a single-component fit. A common issue of these fit-based methods is that their performance is very dependent on the parameter settings, especially the initialization of the quantities to be estimated. Another drawback of fit-based methods is that they perform inferior if more than two lifetime components exist. The objective function becomes more complex if a larger number of lifetime components are fitted, making it extremely difficult to produce an optimal solution. In addition, the optimization of the multiple lifetime components can interact with each other dramatically. It is challenging to ensure an optimal solution for all lifetime components. Therefore, a common understanding is that curve fitting methods in both the global and individual fit regimes are limited to two-component and at maximum three-component analyses.

Alternatively, a pattern-based method was proposed to calculate the abundances based on decay patterns constructed with preselected lifetimes.^20^ Without the need of lifetime estimation, the method can be very fast and capable of multiple-component analysis. However, it is applicable only if the lifetimes are known *a prior* with a high precision. Another option is graphical analysis, which has gained more and more attention as it is a fit-free approach.^21^ While providing excellent and intuitive visualization of lifetimes, graphical analysis does not directly provide the abundance information and its use for quantitative analysis requires calibration samples.^21^

In this contribution, we propose a machine learning (ML)-based method to achieve a fit-free and automatic FLIM data analysis. Herein, the chemometric models were trained with artificial training data and used to predict real-world data. These models took the decay traces as input, and the output was the required lifetimes and abundances. The training data were composed of simulated decay traces with known lifetimes and abundances. The parameters of the simulation, i.e., the number of lifetime components and the range of lifetimes, are predefined according to a rough knowledge of the data to be predicted. The prediction of the testing data is composed mainly of a Laguerre polynomial decomposition (LPD) followed by the prediction with the chemometric models. The approach was verified with both simulated testing data and real-world testing data measured from biological samples. The analysis was conducted for both the two- and three-components tasks. The performance was benchmarked from two aspects: (1) the deviation of the prediction from true values for the simulated testing data and (2) the root-mean-square error (RMSE) between reconstructed and measured decay traces for the real-world datasets. In addition, we compared the ML-based method with the commercial software SPCImage^22^ according to the results of the RMSE.

## 2 Methods and Experiments

Different data sets are used in this work. We utilized artificial generated training data to construct our models. These trained models are evaluated using independently generated artificial test data and independent experimental testing data.

### 2.1 Datasets

#### 2.1.1 Measured testing datasets

The cell data were measured from a single fibroblast cell on the system described in Ref. 23. Briefly, two synchronized picosecond (ps) pulse trains generated by a Ti: sapphire laser (831.7 nm) and an optical parametric oscillator (APE, Germany) (672.3 nm) are focused onto the cell by a 40× objective (C-Apochromat, NA 1.1, Zeiss, Germany). The laser power at the sample was 30 mW at 672.3 nm and 10 mW at 831.7 nm, which ensures the count rate of the FLIM detector below 1 MHz. The fluorescence signal is filtered by a dichroic mirror and two filters (short pass 650 nm, bandpass 458/64  nm, Semrock) and finally is detected by a 2P-FLIM system. The 2P-FLIM system is composed of a hybrid GaAsP detector HPM-100-40 (Becker & Hickl, Germany) and a TCSPC system SPC-150 (Becker & Hickl, Germany). The measurement was done by laser scanning the sample to cover the whole cell area (48.8×48.4  μm) at 12.8  μs pixel dwell time and averaging of 274 frames. In the end, we obtained an FLIM data of 128×128  pixels and 1024 time channels, which spanned the time range of 12.5 ns.

The liver samples were cryosections of the liver specimen of 82-years-old women diagnosed with massively differentiated hepatocellular carcinoma. This specimen was collected during the liver resection operation (right hemihepatectomy) from the area of tumor border. The four FLIM datasets shown in Figs. 3 and 4 and Figs. S4–S9 in the Supplemental Materials were measured at the same setup as described above. The liver tissue specimen is 450  μm in size and measured by a 20× NA 0.8 objective (Planapochromat, Zeiss, Germany) using 512×512  pixel, 1024 time channels, 1.61  μs pixel dwell time, averaging of 95 frames at a laser power of 25 mW at 671.5 nm and 35 mW at 832 nm.

Fetal calf serum (Thermo Fisher Scientific, Waltham, Massachusetts) and pooled human citrated plasma (obtained from the Institute for Transfusion Medicine, Jena University Hospital, Germany) were dripped onto a slide for measurements. The FLIM data (Figs. S11–S14 in the Supplemental Materials) were measured at the setup described above by a 20× NA 0.8 objective (Planapochromat, Zeiss, Germany), with 128×128  pixels, 1024 time channels, 2.56-μs pixel dwell time, and averaging of 100 frames at a laser power of 42 and 54.7 mW at 670.8 nm for the two samples, respectively.

#### 2.1.2 Simulated testing dataset

The simulated data featured a spatial dimension of (128×128) and 921 time channels that are identical with the measured data. The two- and three-components testing data were constructed with the lifetimes and abundances randomly selected within the ranges given in Table S2 in the Supplemental Materials. The abundances of different components were scaled to $\sum_{i=1}^{N}\alpha_{i}=1$. With the broad lifetime ranges, we intended to test if the proposed method is able to handle data featuring different lifetimes without being retrained with new training data. The decays were calculated following Eq. (2), where P represents Poisson noise added to the decay traces. The IRF (R) was taken from the cell data. The number 150 was implemented to simulate the maximal photon counts of 150:

$$I(t)=\int_{0}^{t}R(t-T)\underset{i}{\sum }150\times \alpha_{i}\cdot \exp(-\frac{T}{\tau_{i}})dT+P.$$ (2)

### 2.2 Preprocessing

As the first step of the preprocessing, we cut off the time channels before the 40th bin (photon counting did not start) and after the 960th bin (counting already stopped). This results in 921 time points for each decay trace. The time channel was adjusted to make sure it started with 0 ns. Thereafter, a constant offset representing the dark current response of the detector and ambient light was subtracted from each decay trace. The value of the offset was determined by averaging the time channels preceding the rise of the decay trace. The resulting decay traces were subject to a deconvolution procedure described in the following [see Eq. (3)] and finally an $l_{2}$-norm normalization [Eq. (4)] was applied.

As it is shown in Eq. (1), a measured decay trace is a convolution of the exponential decay with the IRF. A deconvolution is to reverse the convolution and retrieve the real fluorescence decay traces. This was achieved via an LPD as summarized in Eq. (3).^24^^,^^25^ In particular, Eq. (3a) gives the definition of the Laguerre polynomial, with two parameters: the order n and the scale α. The variable x represents the time values in our study. To achieve the deconvolution, the Laguerre polynomial ($B_{n}^{\alpha }$) is first convolved with the IRF following Eq. (3b), where k denotes the index of data points. Thereafter, the decay traces ($I^{m\times p}$) were projected onto the convolved Laguerre polynomials ($L_{n}^{\alpha }$) via a constraint least squares algorithm [Eq. (3c)].^26^ Eventually, the deconvolved decay traces were obtained by multiplying the coefficients matrix ($C^{m\times K}$) with the Laguerre polynomials [Eq. (3d)]. Herein, the term K gives the number of Laguerre polynomials employed. The parameters m and p represent the dimension of the FLIM data, i.e., the number of decay traces and number of time points. Note that we used the continuous instead of the discrete Laguerre polynomials in Refs. 24 and 25, because the latter requires a factorial calculation. A 64-bit computer allows factorial of a number at maximum 150 (k>150), which makes the calculation impossible for data with 921 points in our study (k≤921):

$$B_{n}^{\alpha }(x)=e^{x}\frac{x^{-\alpha }}{n!}\frac{d^{n}}{dx^{n}}(e^{-x}x^{n+\alpha }),\;n=0,1,2,\cdots ,\alpha >-1,$$ (3a)

$$L_{n}^{\alpha }(k)=\overset{k}{\underset{i=1}{\sum }}R(k-i)\cdot B_{n}^{\alpha }(i)=R⊛B_{n}^{\alpha },$$ (3b)

$$I^{m\times p}=C^{m\times K}\times L^{K\times p},$$ (3c)

$$I_{d}^{m\times p}=C^{m\times K}\times B^{K\times p},$$ (3d)

$$I(t_{i})=I(t_{i})/\underset{j}{\sum }I{\left(t_{j}\right)}^{2}.$$ (4)

### 2.3 Modeling and Prediction

The essence of our ML-based approach is the construction of a chemometric model translating the decay traces to the lifetimes and abundances of interest. This requires training data for which the lifetimes and abundances are known exactly and span a broad range covering all values expected from a sample to be predicted. Such training data, however, are extremely difficult to acquire in real-world, due to many restrictions of an FLIM measurement. Foremost, the lifetime is dependent on the microenvironment, which cannot be completely controlled. Moreover, a successful FLIM measurement is possible only if the system matches well to the optimal excitation and emission properties of a fluorophore. This limits the possible fluorophores to be measured on a given system, even for a single-component sample. To measure samples containing multiple lifetime components is a lot more complicated, where we have to guarantee the components do not interact. In addition, the multiple fluorophores have to be carefully selected to avoid FRET. Because of these practical issues, lifetimes are limited to certain values instead of spanning a broad range in a real-world measurement, which is insufficient as training data.

A more feasible and cost-effective solution is to construct artificial training data, which can be generated with a large sample size and over a wide range of lifetimes. Given the lifetime $\tau_{i}$ and abundance $\alpha_{i}$ of each component, we can easily generate a decay trace according to the model in Eq. (5). Thereby, N gives the number of lifetime components within the sample. P represents Poisson noise added to the decay traces. The lifetime $\tau_{i}$ and abundance $\alpha_{i}$ of the i’th component in each decay trace are randomly selected from predefined ranges $\left[\tau_{i}^{l},\tau_{i}^{h}\right]$ and $\left[a_{i}^{l},a_{i}^{h}\right]$. The abundances of different components are rescaled to unit sum before the decay trace is generated. The values of N, $\tau_{i}^{l}$, $\tau_{i}^{h}$, $a_{i}^{l}$, and $a_{i}^{h}$ can be determined according to *a prior* knowledge of the samples of interest. This property contributes to the flexibility of the method to be tailored for different studies. Given the range of the lifetime and abundance is sufficiently broad, a model is supposed to be usable for multiple studies without retraining. Noteworthy, the training data are simulated as exponential decays without any convolution to IRF. The idea is to make sure the trained models are independent of the measurement system (i.e., IRF) and able to be used among different measurement systems:

$$I(t)=150\times \overset{N}{\underset{i}{\sum }}\alpha_{i}\;\exp(-\frac{t}{\tau_{i}})+P,\;const.\;\tau_{i}\in [\tau_{i}^{l},\tau_{i}^{h}];\;\alpha_{i}\in [a_{i}^{l},a_{i}^{h}];\;\overset{N}{\underset{i=1}{\sum }}\alpha_{i}=1.$$ (5)

With the training data available, we are ready to build 2N chemometric models, each responsible for the lifetime or abundance of a different component. Training a different model separately for each quantity (i.e., lifetime or abundance) allows us to extract their values independently. The models all shared the same structure and hyperparameters: the decay traces were subject to a principal component analysis (PCA) for dimension reduction, following a random forest-based model using the first 15 PCA components. The random forest was composed of 500 trees, each tree trained with 63.2% of training data based on 12 randomly selected features (parameters ntree=500, samplesize=0.632, and mtry=12 in the R package of “RandomForest”^27^). This bootstrapping-based training procedure is the standard training procedure for random forests, but it is not related to the training-testing data split but represents a local separation (for every tree) into training and validation data. The model is trained based on this procedure and then applied to independent data (either experimental data or artificial testing data). A random forest was applied, as the model does not make assumptions about the data being modeled.

After being trained, the chemometric models are ready to predict the real-world data. To start, the FLIM data is preprocessed with previously described steps. The prediction of the model is then conducted on the resulting deconvolved decay traces, with the lifetimes and abundances of all components as outputs. The predicted abundances $\alpha_{i}$ are then rescaled as $\sum_{i=1}^{N}\alpha_{i}=1$. The results of the lifetimes and abundances can then be stored and visualized for further interrogation by biologists or physicians.

## 3 Results and Discussion

Following the description of the workflow of our method, we now move to the results in this section. Herein, the analysis was carried out for both the two- and three-component modeling using the simulated and the measured test datasets. To start, the chemometric models were trained with 3000 decay traces constructed with two or three lifetime components, respectively. The parameters of the training data, $\tau_{i}^{l}$, $\tau_{i}^{h}$, $a_{i}^{l}$, and $a_{i}^{h}$, are summarized in Table S1 in the Supplemental Materials. These values were chosen based on three considerations. Foremost, the lifetime of a biological autofluorophore, which is of top interest in our daily research, is usually no larger than 7 ns.^28^ Moreover, we aimed to span as broad range of lifetimes as possible in the training data so that the models can predict different samples with minimal requirement of retraining. In addition, the lifetime ranges of different components partly overlap. This allows the model to resolve different components even if their lifetimes are close to each other.

After the training procedure, the models are applied to predict multiple testing samples without retraining. To do so, the testing data were first preprocessed and deconvolved with the LPD method to retrieve the exponential decays. In particular, the LPD was conducted based on 15 Laguerre polynomials (α=2, n=1:15). These polynomials and the results from the convolution with the IRF of the data to be processed are shown in Figs. S1(a) and S1(b) in the Supplemental Materials. The parameters of the Laguerre polynomials were determined by an trial-and-error to make sure that the decay traces can be well reconstructed from C×L. This is an important prerequisite to successfully derive the lifetimes in the next step. The means of the reconstructed and the raw decay traces are given in Figs. S1(c) and S2 in the Supplemental Materials for all datasets. In summary, all traces show good fit quality. The mean of the deconvolved decay for the cell data can be found in Fig. S1(d) in the Supplemental Materials. Noteworthy, the IRF used in our study was exported for each dataset from the software SPCImage. It can be replaced in real-world applications with a fluorescence decay recorded from an infinitely short fluorescence lifetime.

The decay traces after deconvolution were fed into the chemometric models to obtain the lifetimes and abundances. The results are presented in the following for both the simulated and measured testing datasets. The performance of the analysis was verified from two aspects. On one hand, the predicted quantities were compared with their true values for the simulated testing data, which acted as the basic benchmark of the models. On the other hand, we compared the analysis of the real-world data with that of the SPCImage, which was performed on the time channels 40-960, with either two- or three-component fit. To do so, we reconstructed the decay traces following Eq. (1) using the predicted quantities for the ML method and the exported lifetimes ($\tau_{i}$) and abundances in percentage ($\alpha_{i}\%$) for the SPCImage. The reconstruction was benchmarked with the RMSE defined by Eq. (6), which characterizes the derivations between the reconstructed (I^) and measured decay traces (I). In the next sections, we will first present the results on simulated data, following those on the real-world data:

RMSE=1n∑i=1n(Ii−I^i)2. (6)

### 3.1 Prediction on Simulated Testing Data

**Fig. 1:**
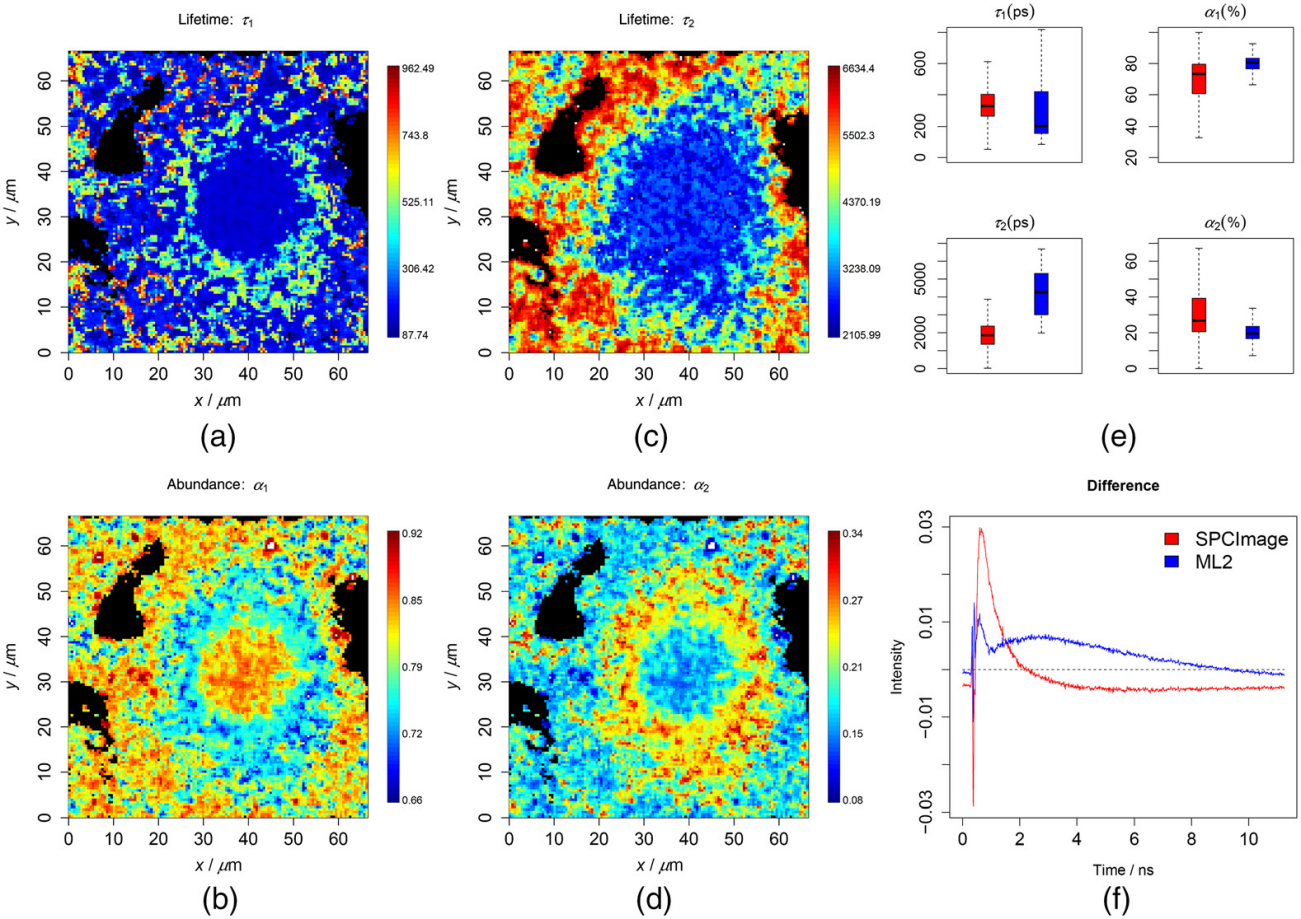
Results of the two-component analysis of the cell testing data. To ensure a good contrast, all false-color plots were generated based on 0.001 to 0.999 percentiles of the values to be visualized. (a)–(d) Lifetimes and abundances of the two components. (e) Results of the ML method (blue) along with the SPCImage (red), which are generally similar but do not match exactly. (f) Difference between the means of the reconstruction and the raw data. The RMSE was 0.452 and 0.474 for the ML and SPCImage, respectively.

As the first verification, the chemometric models were used to predict the simulated testing data. The differences between the true and predicted values are shown as boxplots in Figs. S3(a) and S3(c) in the Supplemental Materials. The average values are shown to be around zero in all cases, which means an unbiased prediction. In the same time, the confidence interval (CI) was ±10∼15% for the lifetimes (CI/t%) and ±5∼10% for the abundances (CI%). This means the models were able to extract the quantities of the two lifetime components satisfactorily. The predicted results also allowed us to retrieve the decay traces following Eq. (1), as it was demonstrated by the means of the reconstructed and the raw traces in Figs. 1(b) and 1(d). These results indicate a high possibility of the ML method to predict datasets of various lifetimes without being retrained on new training data.

### 3.2 Real-World Testing Data

**Fig. 2:**
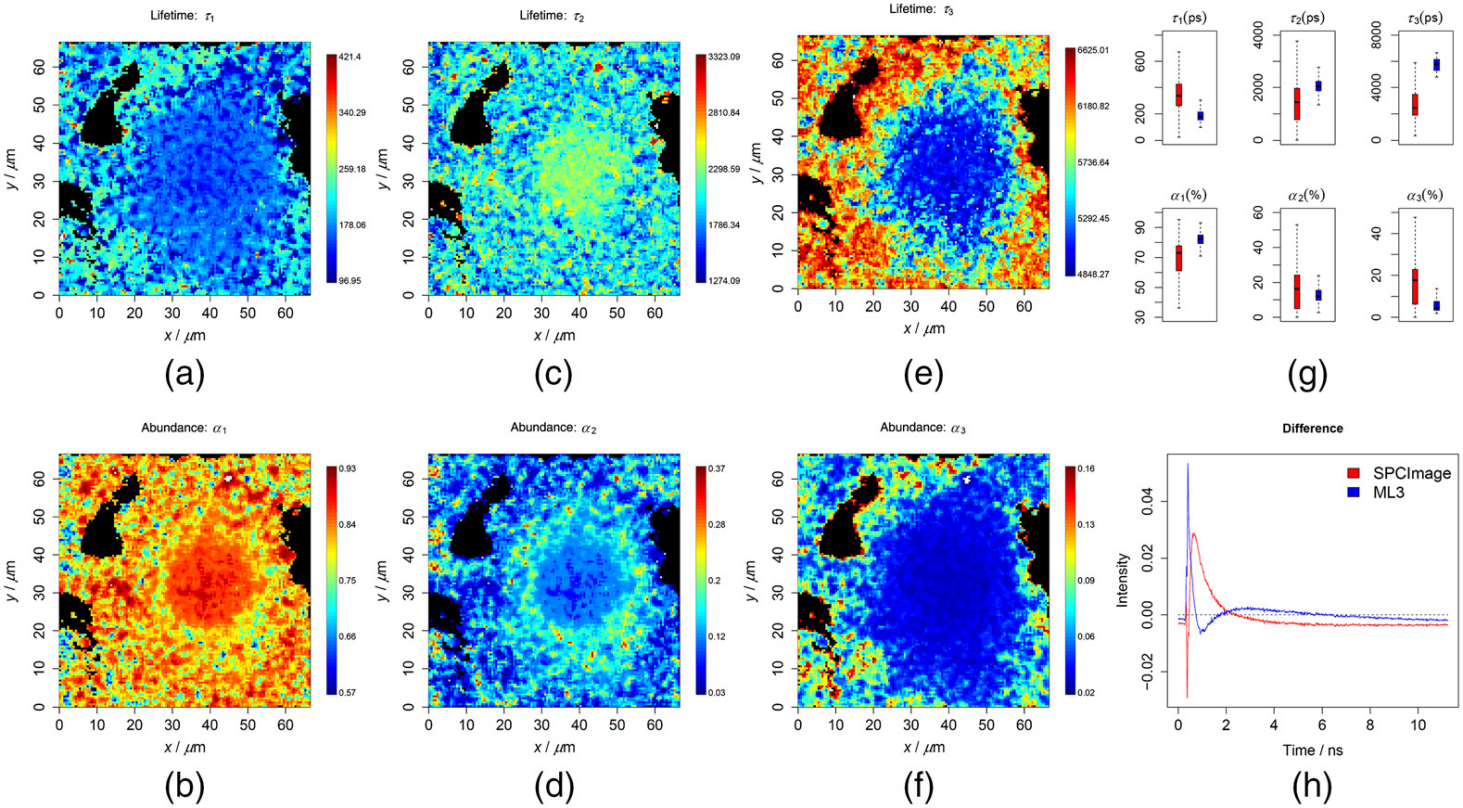
Results of the three-component analysis on the cell testing data. To ensure a good contrast, all false-color plots were generated based on 0.001 to 0.999 percentiles of the values to be visualized. (a)–(f) Lifetimes and abundances of the three components. (g) Results of the ML method (blue) along with the SPCImage (red), which are generally similar but do not match exactly. (h) Difference between the means of the reconstruction and the raw data. The RMSE was 0.439 and 0.474 for the ML and SPCImage, respectively.

**Fig. 3:**
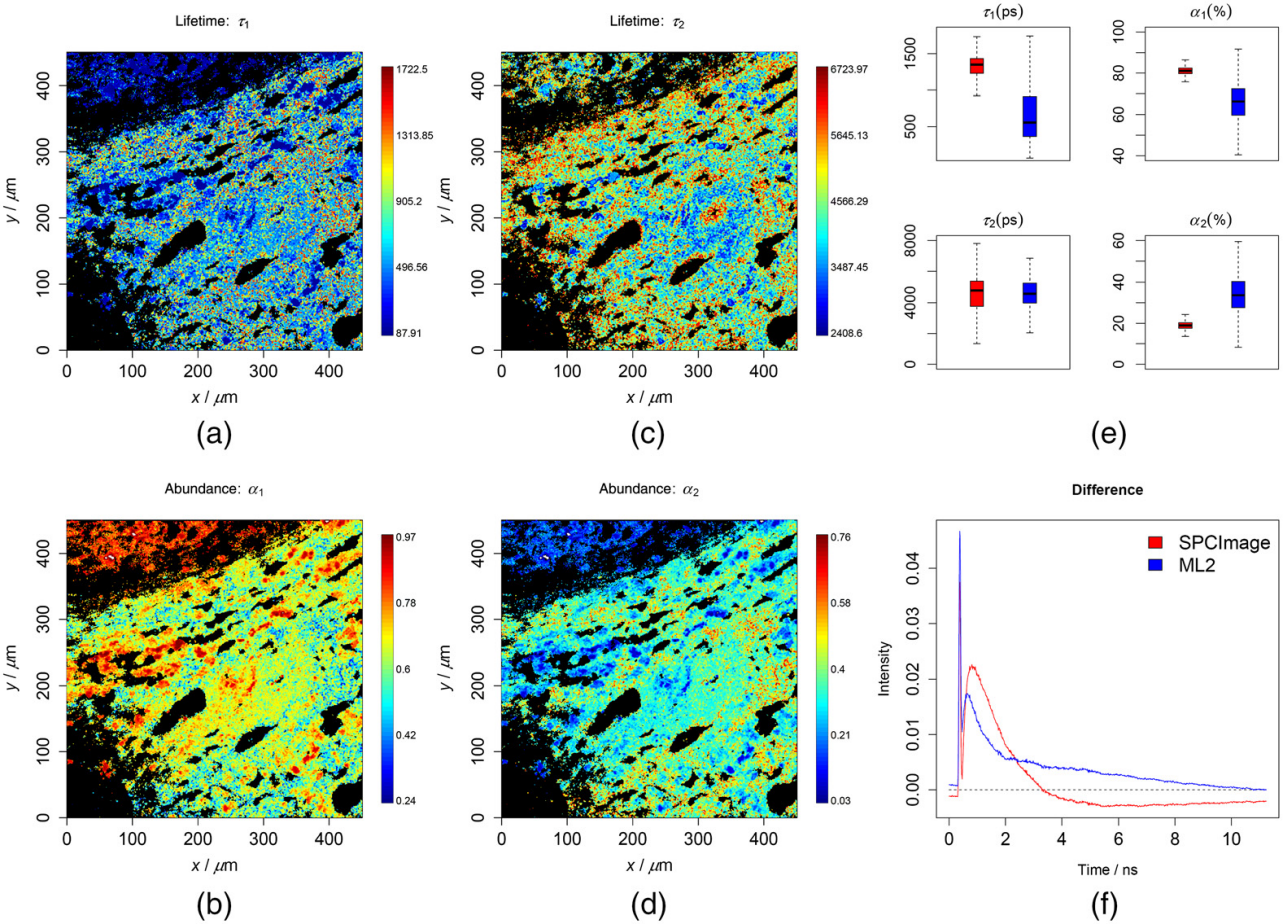
Results of the two-component analysis of the first liver tissue testing data. To ensure a good contrast, all false-color plots were generated based on 0.001 to 0.999 percentiles of the values to be visualized. (a)–(d) Lifetimes and abundances of the two components. (e) Results of the ML method (blue) along with the SPCImage (red), which are generally similar but do not match exactly. (f) Difference between the means of the reconstruction and the raw data. The RMSE was 0.577 and 0.602 for the ML and SPCImage, respectively.

**Fig. 4:**
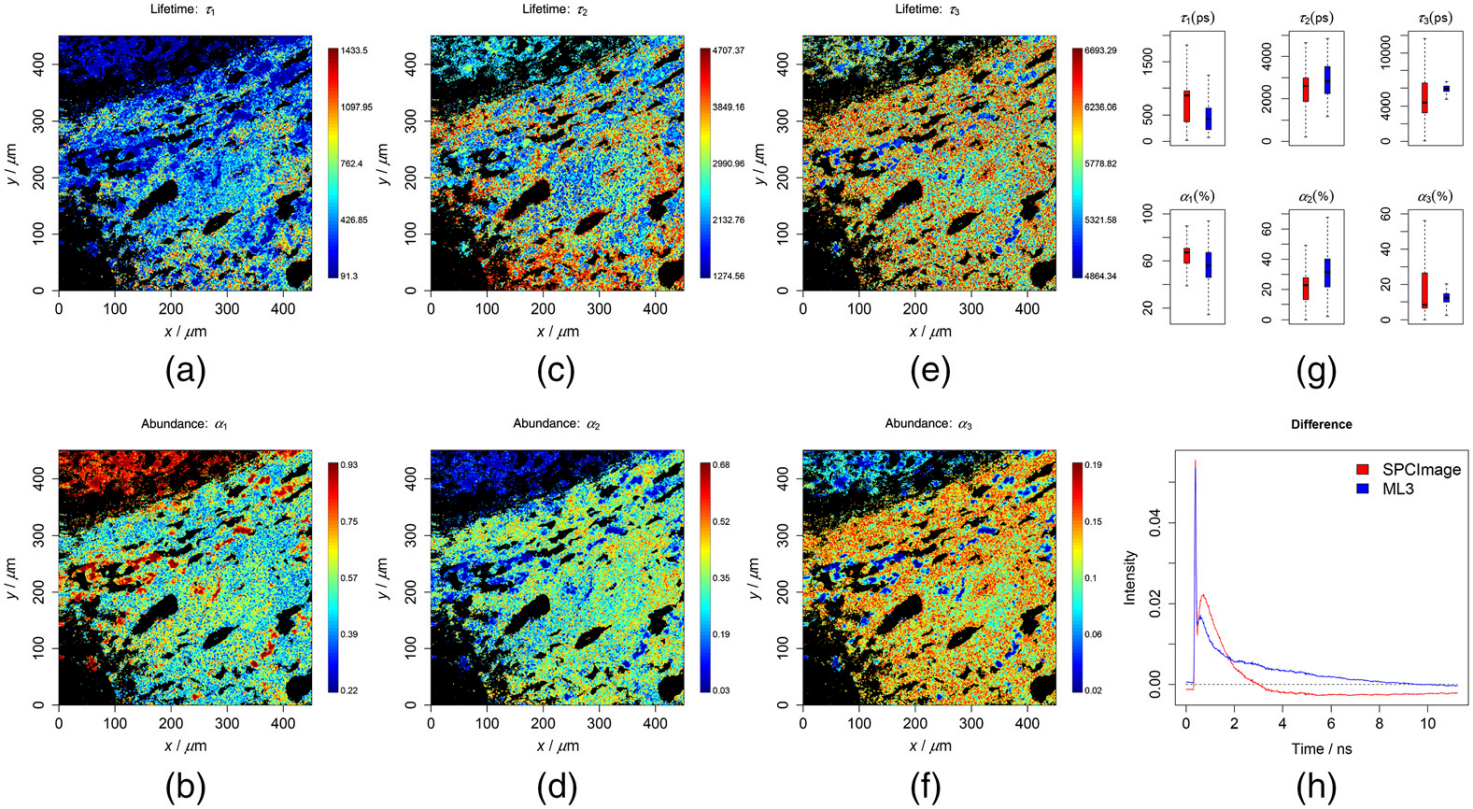
Results of the three-component analysis of the first liver tissue testing data. To ensure a good contrast, all false-color plots were generated based on 0.001 to 0.999 percentiles of the values to be visualized. (a)–(f) Lifetimes and abundances of the two components. (g) Results of the ML method (blue) along with the SPCImage (red), which are generally similar but do not match exactly. (h) Difference between the means of the reconstruction and the raw data. The RMSE was 0.576 and 0.602 for the ML and SPCImage, respectively.

For a further verification, the chemometric models were used to predict measured data after being preprocessed by the previously mentioned steps. The results from the cell data are shown in Figs. 1 and 2 in the cases of the two and three components, respectively. The lifetimes and abundances are shown with the unit of ps and percentage (%), respectively. The regions in black are excluded from the analysis, where the maximum photon counts of the decay trace were below 9. These regions did not contain cell information and represent mostly the substrate. The first two lifetimes were found at around 200 to 300 and 2000 to 3000 ps, respectively, and were located at the central region of the image where the cell is located. These are in line with *a prior* knowledge on the lifetimes of free and protein-bound NAD(P)H. In Figs. 1(e) and 2(g), the predicted lifetimes were visualized as box plots in blue color along with the results from the SPCImage analysis in red color. Therein, we could see that the results match roughly but not exactly. To make a more conclusive comparison, we additionally reconstructed the decay traces based on the resulting quantities from the ML method and SPCImage. The average differences between the reconstruction and the true decay traces are given in Figs. 1(f) and 2(h) for the two methods in two- and three-component analyses, respectively. The reconstruction error (RMSE) was observed lower for the ML analysis, especially in the case of the three-component analysis. This indicates most probably that the ML method fits better to the decay traces than the SPCImage analysis. A similar conclusion could be drawn from the results of the liver tissue as shown in Figs. 3 and 4 and Figs. S4–S9 in the Supplemental Materials. In these plots, the lifetimes of 200 to 300 and 2000 to 3000 ps are expected for free and protein-bound NAD(P)H. The black regions shown in the images are excluded from the analysis as the maximal photon counts of the decay traces are below 5. The results from ML method are consistent with *a prior* knowledge both in two- and three-component analyses.

In Figs. 1–4 and Figs. S4–S9 in the Supplemental Materials, it can be seen that the values of the first two lifetimes in the three-component analysis are closer to their expected results, 200 to 300 and 2000 to 3000 ps for free and protein-bound NAD(P)H, respectively. This shows to a large extent that the different lifetime components were better resolved by additionally including a slower component as the third component, which indicated that these samples very likely contain a third lifetime component. This is different in the cases of the FSC and plasma datasets. According to the results in Figs. S11–S14 in the Supplemental Materials, the results are better in line with *a prior* knowledge of the lifetime range (200 to 300 and 2000 to 3000 ps) for the two-component analysis.

**Fig. 5:**
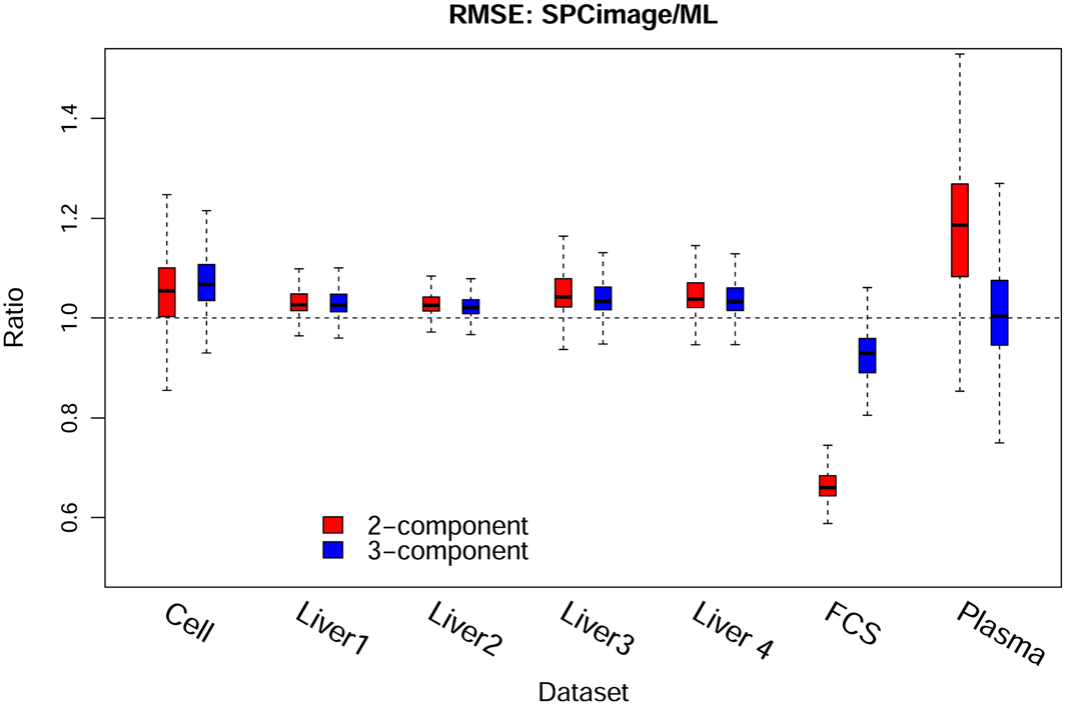
RMSE ratios between the SPCImage and ML analysis calculated from each decay trace separately. Boxes in red and blue represent the results of two- and three-component analyses, respectively. The ratios are generally above 1 except for the FSC data, indicating a better performance of the ML method than SPCImage.

To make the above discussions more quantitative, we calculated the RMSE of each decay trace between the reconstruction and the raw data for both methods, e.g., ML and SPCImage. The results are shown in Fig. S10 in the Supplemental Materials, in which each subplot corresponds to one dataset. The labels on the x axis, “SPC2,” “SPC3,” “ML2,” and “ML3” specified the cases of two- and three-component analyses by SPCImage and ML method, respectively. The medians are summarized in Table 1, where a smaller RMSE was observed for ML than for SPCImage in all cases but the FSC data. In particular, lower RMSEs were seen in the cell and liver data for the three-component analysis in comparison with the two-component analysis. This again demonstrated the fact that the former five datasets are more likely three-component systems while the latter two samples (FSC and plasma data) are two-component systems. We visualized additionally the ratio of each decay trace between the RMSE of SPCImage and ML method in Fig. 5. The dash line in Fig. 5 marked the ratio of 1, i.e., equal RMSE for the two methods. The ratios are mostly above 1, which demonstrated again a lower RMSE for the ML than the SPCImage analysis.

**Table 1:** The median RMSE (ϵ) between the raw and reconstructed decay traces for all datasets in the cases of SPCImage and ML analysis. The RMSE for the ML method is generally smaller than for the SPCImage-based results.

	Cell	Liver 1	Liver 2	Liver 3	Liver 4	FSC	Plasma
ML2	0.465	0.584	0.529	0.588	0.598	0.141	0.108
ML3	0.450	0.562	0.517	0.559	0.597	0.120	0.121
SPC2	0.476	0.586	0.537	0.596	0.632	0.093	0.126
SPC3	0.473	0.566	0.520	0.562	0.623	0.110	0.123

Before ending this section, it is worth to mention another work published lately,^29^ in which a fit-free analysis of two lifetime components was achieved by a 3-D convolutional network named FLI-Net. In comparison with FLI-Net, our method is meant to push the fit-free FLIM analysis further in three aspects. First of all, the analysis was conducted on each decay trace separately; thus the method has no constraint on the spatial dimension of the data and can be used without retraining for data of different spatial dimensions. Moreover, we have largely minimized the need of model retraining encountering different datasets/measurements. We were able to analyze all the datasets, both simulated and measured, using the same chemometric models without retraining. This was ensured by the LPD procedure, which makes the ML method independent of IRF and paves the way to cross-system/measurement prediction of the models without retraining. In addition, the parameters for the training data were made to cover a broad range of lifetimes, which helps the ML method to generalize to datasets featuring different lifetimes. The prediction on the simulated testing data, which contains lifetimes of large ranges, well demonstrated the generalization of the ML method without retraining. Nonetheless, a retraining will be necessary if the lifetime range of the training data do not well represent the expected lifetimes, i.e., training data with different $\tau_{i}^{l}$, $\tau_{i}^{h}$ would be required. Furthermore, we extended the analysis to more than two lifetime components, demonstrated by the good results of the three-component task.

### 3.3 Time Considerations

Besides the goodness of the reconstruction, another important criterion for FLIM analysis is the time required for the analysis. In our method, the time is consumed mainly by the LPD deconvolution and the prediction of the chemometric models. Accordingly, we recorded the time required after importing data to the end of the prediction for all testing datasets. In comparison, the time required for SPCImage was recorded on the same computer. The results are summarized in Table 2. The terms “ML2” and “ML3” represent the two- and three-component analyses, respectively. The ML method takes longer than SPCImage. However, it should be noted that the analysis was simply done on CPU based on Microsoft Open R at this concept-proof stage. As both the LPD and the prediction can be easily parallelized, we see a large space to speed up the method and will proceed this in the near future.

**Table 2:** Time (t, in seconds) required for the analysis of different datasets. The ML method in current stage takes longer than SPCImage. However, we expect to largely reduce the computation of ML method in the next step.

	Cell	Liver 1	Liver 2	Liver 3	Liver 4	FSC	Plasma
ML2	24	304	374	337	285	25	25
ML3	25	324	400	356	331	26	25
SI2	13	76	99	77	74	13	13
SI3	18	85	123	98	85	18	18

## 4 Conclusion

We demonstrated in this contribution an ML-based method for a fit-free and automatic analysis of FLIM data. In combination with an LPD deconvolution, we could make the method independent to the measurement system and able to be transferred across measurements. We verified the method on both simulated and measured datasets. The prediction was proven to match well with the true values for both two- and three-component analyses according to the simulated testing data. As the simulated testing data featured lifetimes of broad range, the satisfactory prediction also illustrated the generalizability of the model to data of different lifetimes without retraining. In addition, the comparison between the ML and the SPCImage methods showed that the ML method gives smaller RMSE between the reconstructed and the raw decay traces. The improvement of the fits is in the range of 5%, which might be important in clinical scenarios where the difference between, e.g., inflammation and cancer growth is often extremely small. Any improvement in the data processing may lead to better and more stable diagnostics. In addition, the improvement of the reconstruction from SPCImage to ML is more pronounced for decays with higher noise, as is shown in Fig. S15 in the Supplemental Materials. This means that our ML method is better suited to handle noisy datasets, which is often encountered in clinical data. Furthermore, the satisfactory results in three-component analysis showed high potential of the method in FLIM data of multiple-components, which is necessary to push FLIM to more complex applications.
