# Supplementary material for: FLIM data analysis based on Laguerre polynomial decomposition and machine-learning
Source: J Biomed Opt. 2021 Jan 7;26(2):022909. doi: 10.1117/1.JBO.26.2.022909 (PMC7790506; doi:10.1117/1.JBO.26.2.022909)
Supplement: Supplementary file 1 [file JBO_026_022909_SD001.pdf]

FLIM Data Analysis Based on Laguerre Polynomial Decomposition and Machine-Learning

Shuxia Guo, Anja Silge, Hyeonsoo Bae, Tatiana Tolstik,
Tobias Meyer, Michael Schmitt, Jürgen Popp, Thomas Bocklitz*

1. Institute of Physical Chemistry and Abbe Center of Photonics, University of Jena, 07743 Jena, Germany
2. Leibniz Institute of Photonic Technology, Member of Leibniz Health Technologies, 07745 Jena Germany
3. Jena University Hospital, 07743 Jena Germany

* thomas.bocklitz@uni-jena.de

Table S1. Parameters used for generating the training data with the unit of nanosecond (ns) and percentage for the lifetime and the abundance, respectively. Herein 7 ns was used as the upper limit of the lifetimes, as the lifetime of biological autofluorophores is usually not larger than 7 ns. Noteworthy, the abundance values were generated in a way that $\sum_{i=1}^{N} \alpha_i = 100$ % holds true.

	τ_1^l, τ_1^h	α_1^l, α_1^h	τ_2^l, τ_2^h	α_2^l, α_2^h	τ_3^l, τ_3^h	α_3^l, α_3^h
$N = 2$	0.05, 2	0, 100	1, 7	0, 100	--	--
$N = 3$	0.05, 2	0, 100	1, 5	0, 100	4, 7	0, 0.2

Table S2. Parameters used for generating the simulated testing data with the unit nanosecond (ns) and percentage for the lifetime and the abundance, respectively. The abundance values were generated in a way that $\sum_{i=1}^{N} \alpha_i = 100$ % holds true.

	τ_1^l, τ_1^h	α_1^l, α_1^h	τ_2^l, τ_2^h	α_2^l, α_2^h	τ_3^l, τ_3^h	α_3^l, α_3^h
$N = 2$	0.1, 2	0, 100	1, 7	0, 100	--	--
$N = 3$	0.1, 2	0, 100	1, 5	0, 100	4, 7	0, 20

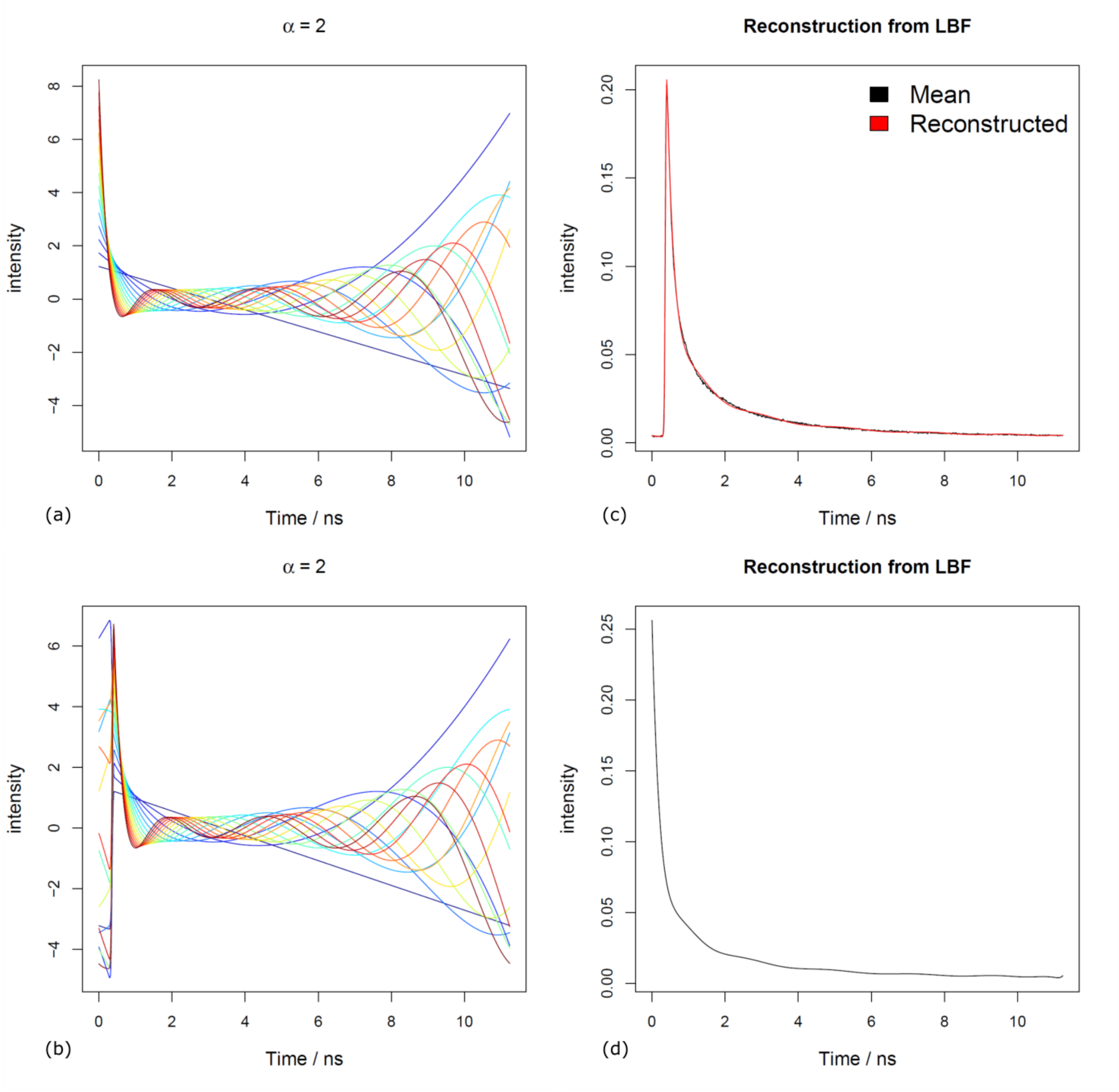

Figure S1. Results of LBD deconvolution. (a) Laguerre basis parameterized by $\alpha = 2, \mathrm{n} = 1:15$. (b) Laguerre basis convolved with IRF. (c) Means of reconstructed decays ($\mathbf{C} \times \boldsymbol{\mathcal{L}}$) and raw decay trace for the cell data. (d) Mean of deconvolved decay trace for the cell data.

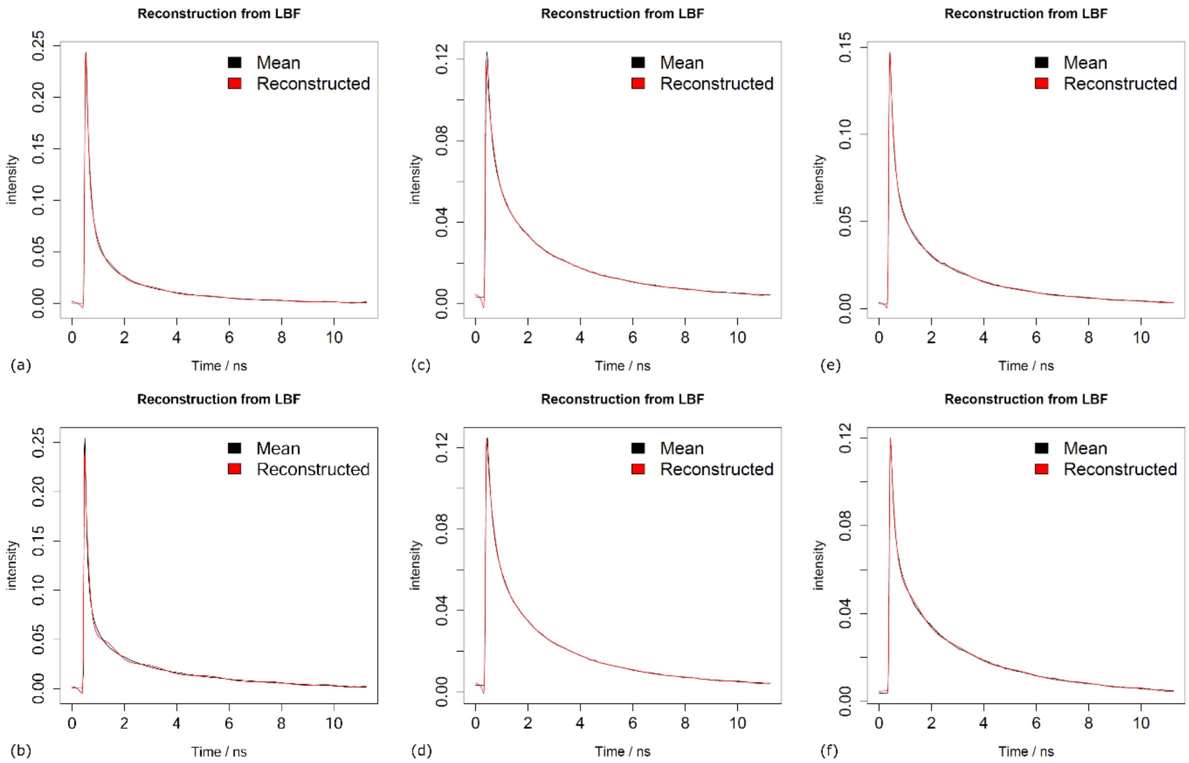

Figure S2: Results of the mean decay traces reconstructed from the Laguerre basis function for the six measured FLIM datasets. (a) FCS; (b) plasma; (c-f) liver tissue

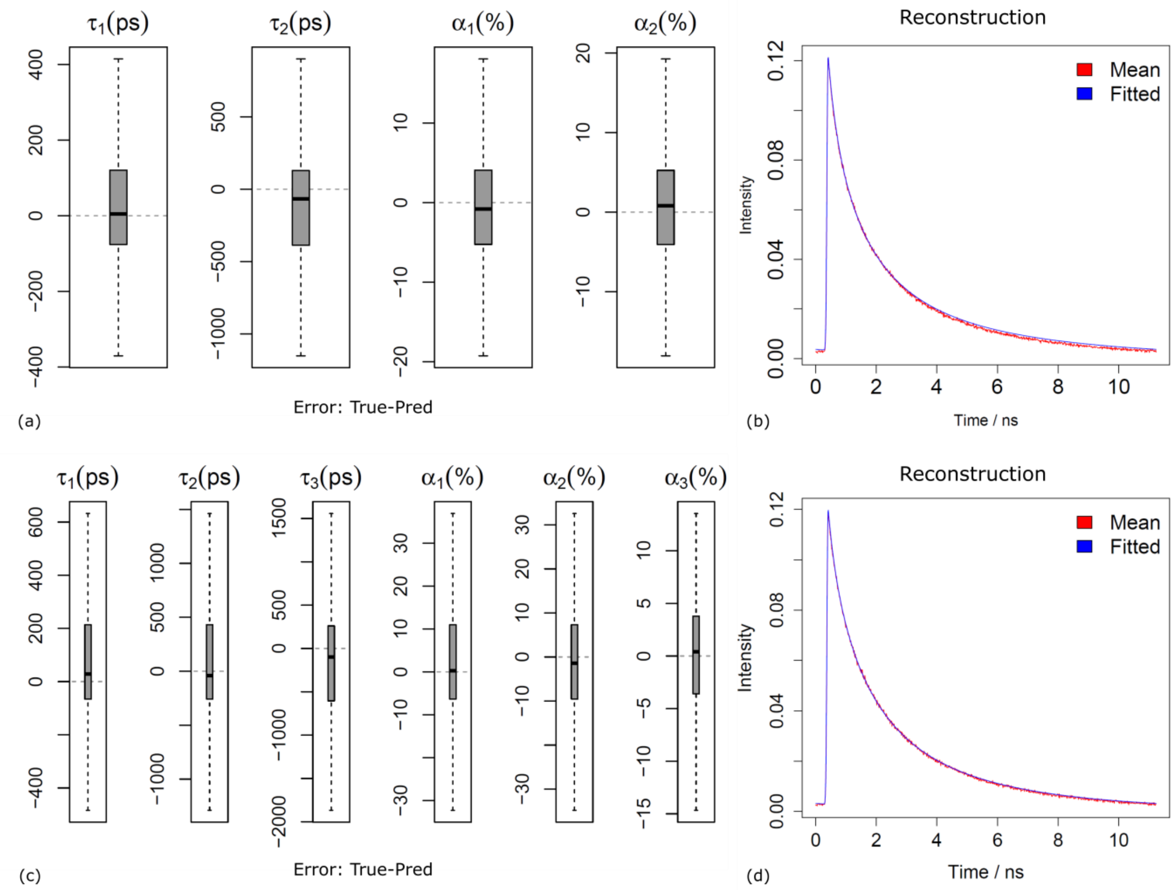

Figure S3: Results of simulated testing data. (a, c) The difference between the true and predicted values for the lifetimes and abundances of different components are shown. Each box contains results of all decay traces. These results indicate a good match between the predicted and the true parameters. (b, d) The mean decay traces of the raw data and the reconstructed results based on the predicted lifetimes and abundances for the 2- and 3-component analysis are plotted. The decay traces are well retrieved using the prediction of the ML method.

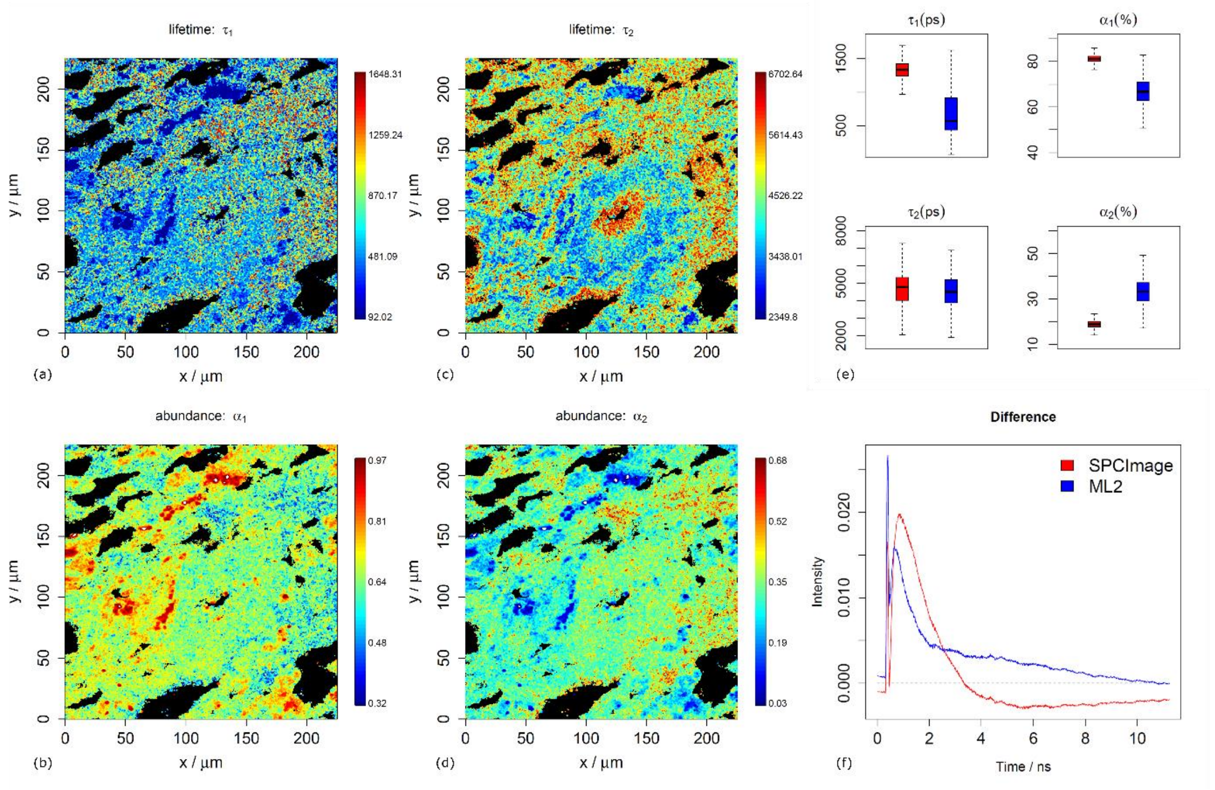

Figure S4. Results of the 2-component analysis on the second liver tissue data. To ensure a good contrast, all false-color plots were generated based on 0.001~0.999 percentiles of the values to be visualized. (a-d) Lifetime and abundance of the two components. (e) Results of the ML method (blue) along with the results of SPCImage (red). (f) Difference between the means of the reconstruction and the raw data.

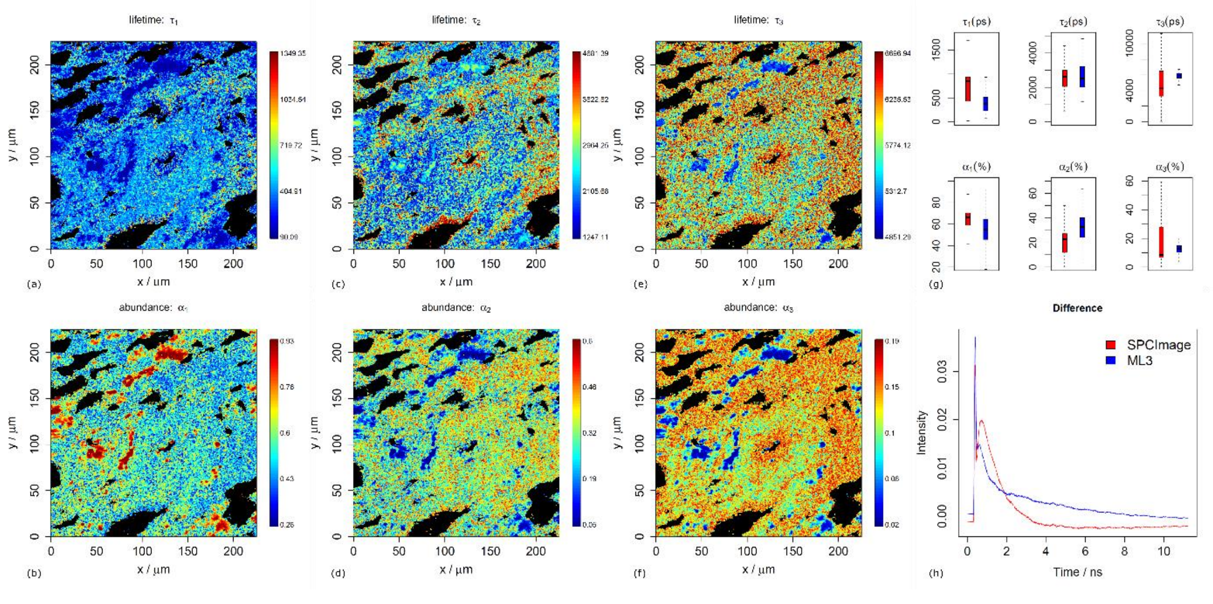

Figure S5. Results of the 3-component analysis on the second liver tissue data. To ensure a good contrast, all false-color plots were generated based on 0.001~0.999 percentiles of the values to be visualized. (a-f) Lifetime and abundance of the three components. (g) Results of the ML method (blue) along with the results of SPCImage (red). (h) Difference between the means of the reconstruction and the raw data.

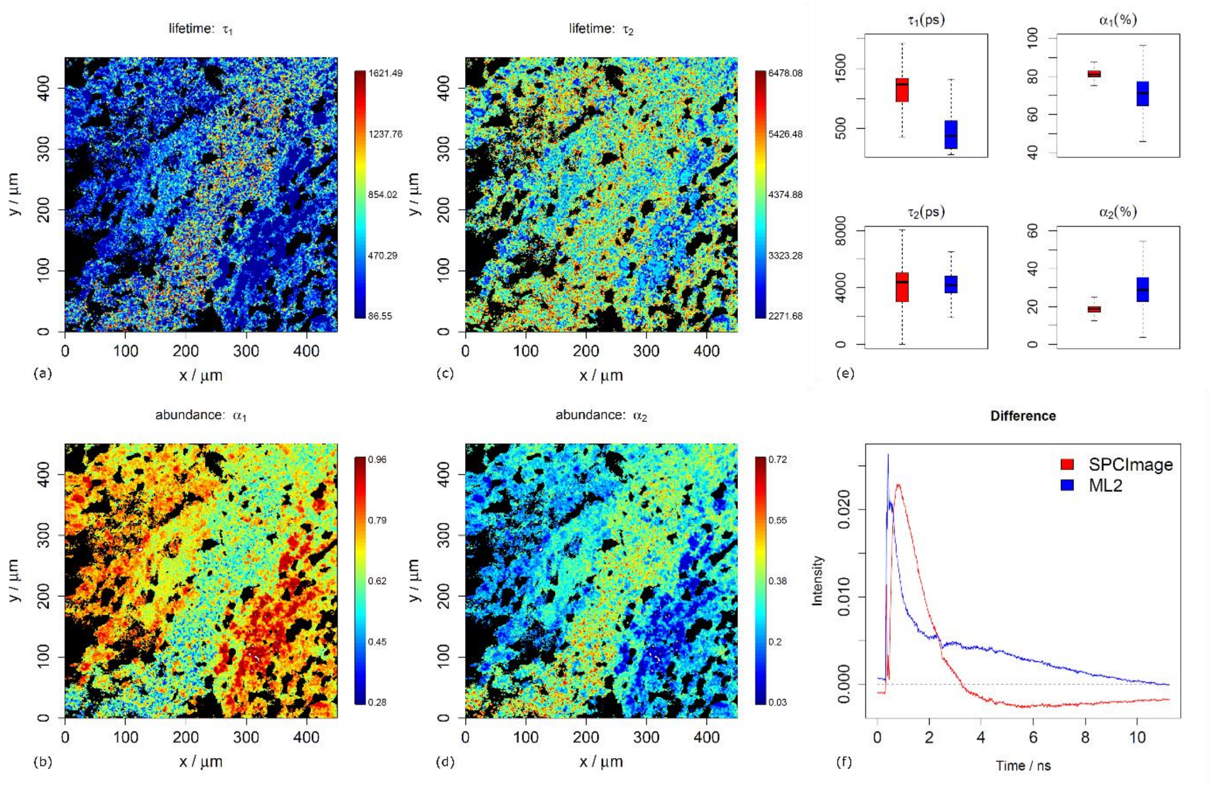

Figure S6. Results of the 2-component analysis on the third liver tissue data. To ensure a good contrast, all false-color plots were generated based on 0.001~0.999 percentiles of the values to be visualized. (a-d) Lifetime and abundance of the two components. (e) Results of the ML method (blue) along with the results of SPCImage (red). (f) Difference between the means of the reconstruction and the raw data.

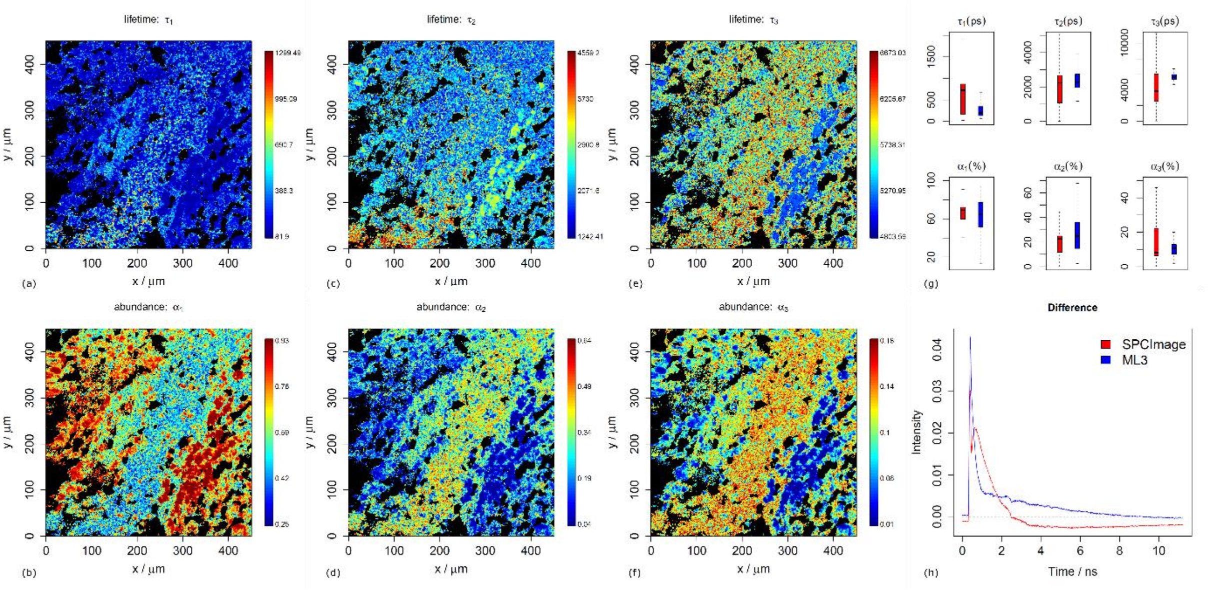

Figure S7. Results of the 3-component analysis on the third liver tissue data. To ensure a good contrast, all false-color plots were generated based on 0.001~0.999 percentiles of the values to be visualized. (a-f) Lifetime and abundance of the three components. (g) Results of the ML method (blue) along with the results of SPCImage (red). (h) Difference between the means of the reconstruction and the raw data.

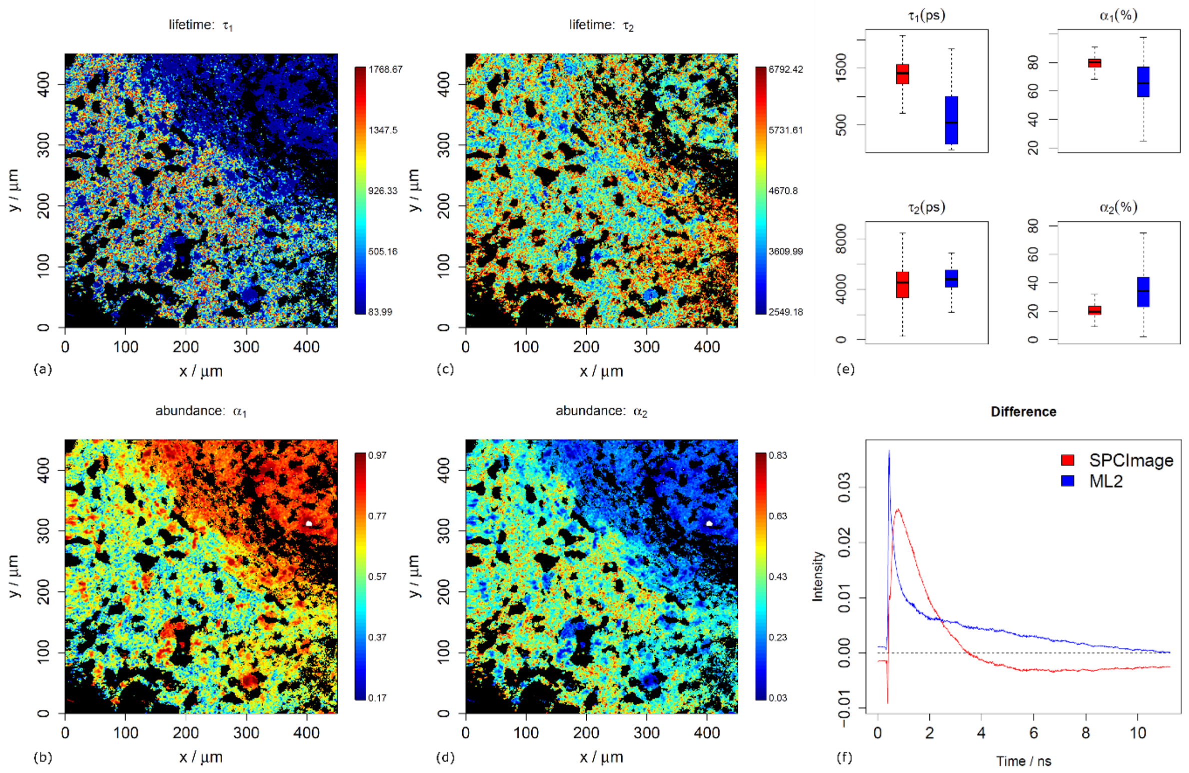

Figure S8. Results of the 2-component analysis on the 4th liver tissue data. To ensure a good contrast, all false-color plots were generated based on 0.001~0.999 percentiles of the values to be visualized. (a-d) Lifetime and abundance of the two components. (e) Results of the ML method (blue) along with the results of SPCImage (red). (f) Difference between the means of the reconstruction and the raw data.

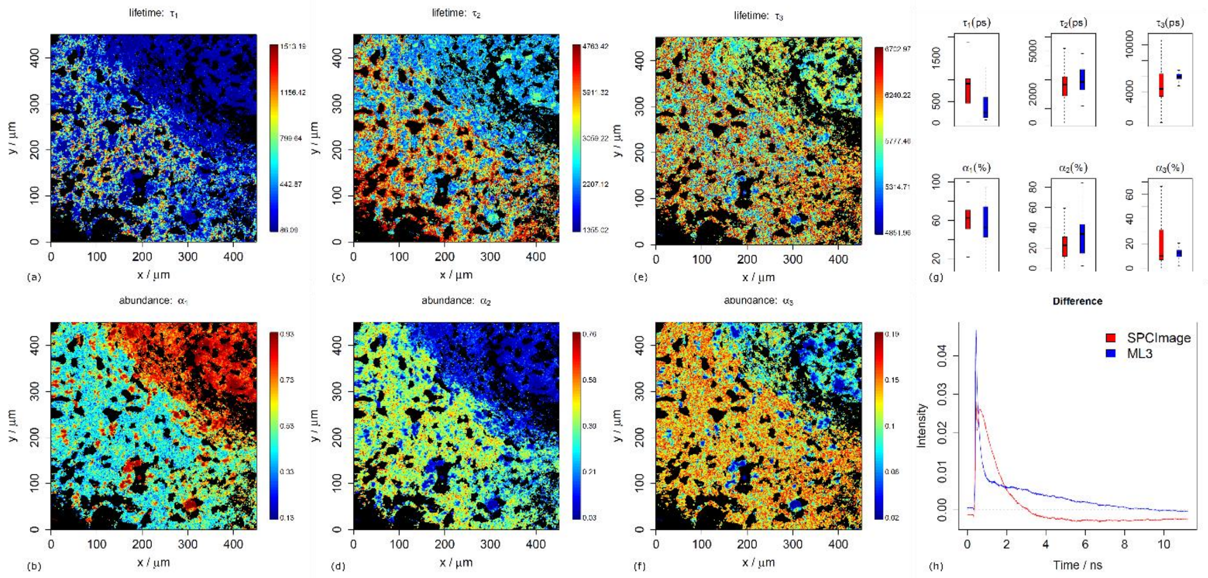

Figure S9. Results of the 3-component analysis on the 4th liver tissue data. To ensure a good contrast, all false-color plots were generated based on 0.001~0.999 percentiles of the values to be visualized. (a-f) Lifetime and abundance of the three components. (g) Results of the ML method (blue) along with the results of SPCImage (red). (h) Difference between the means of the reconstruction and the raw data.

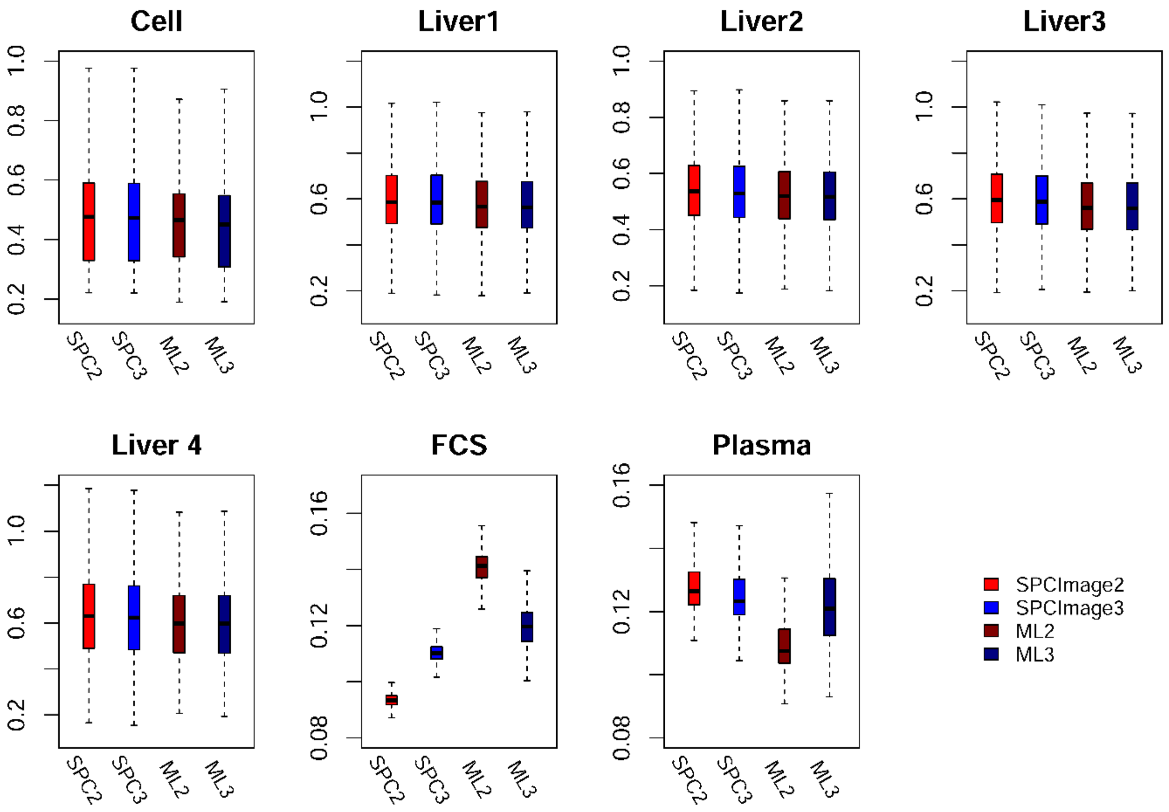

Figure S10 RMSE between reconstructed and raw decay traces in cases of the 2- and 3-component analysis using SPCImage and the machine learning (ML) method.

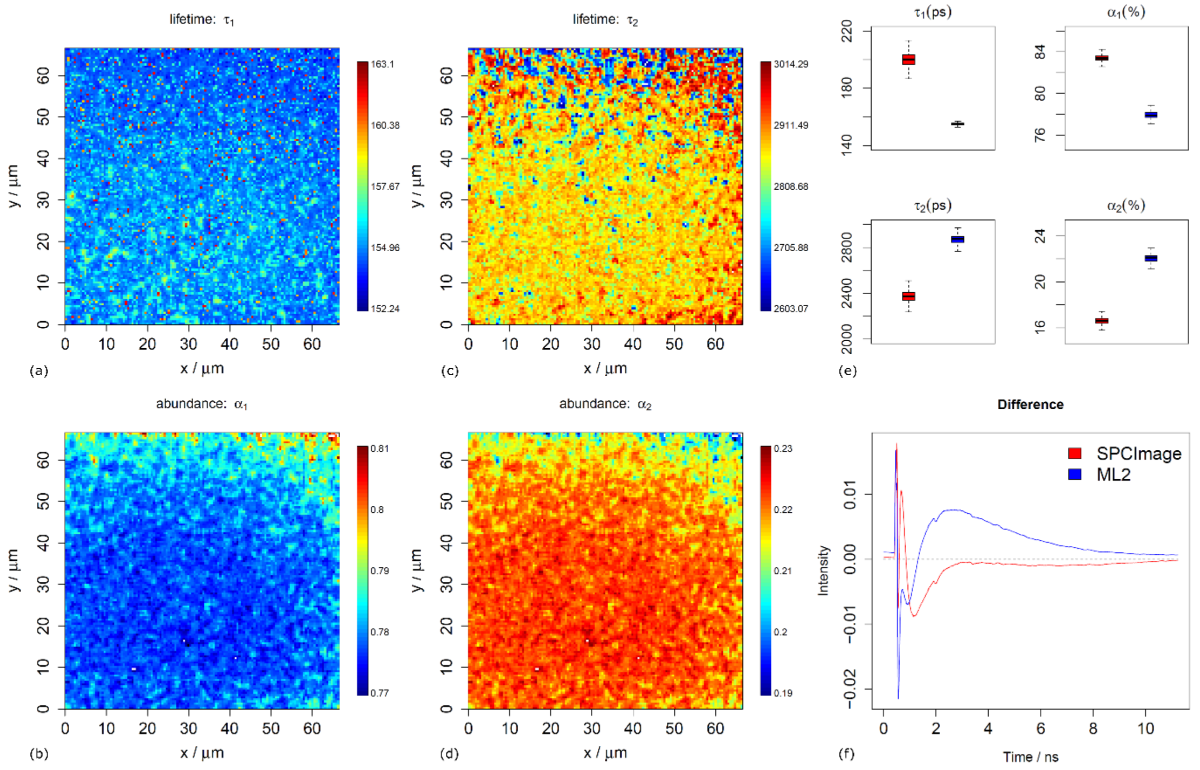

Figure S11. Results of the 2-component analysis on FCS data. To ensure a good contrast, all false-color plots were generated based on 0.001~0.999 percentiles of the values to be visualized. (a-d) Lifetime and abundance of the two components. (e) Results of the ML method (blue) along with the results of SPCImage (red). (f) Difference between the means of the reconstruction and the raw data.

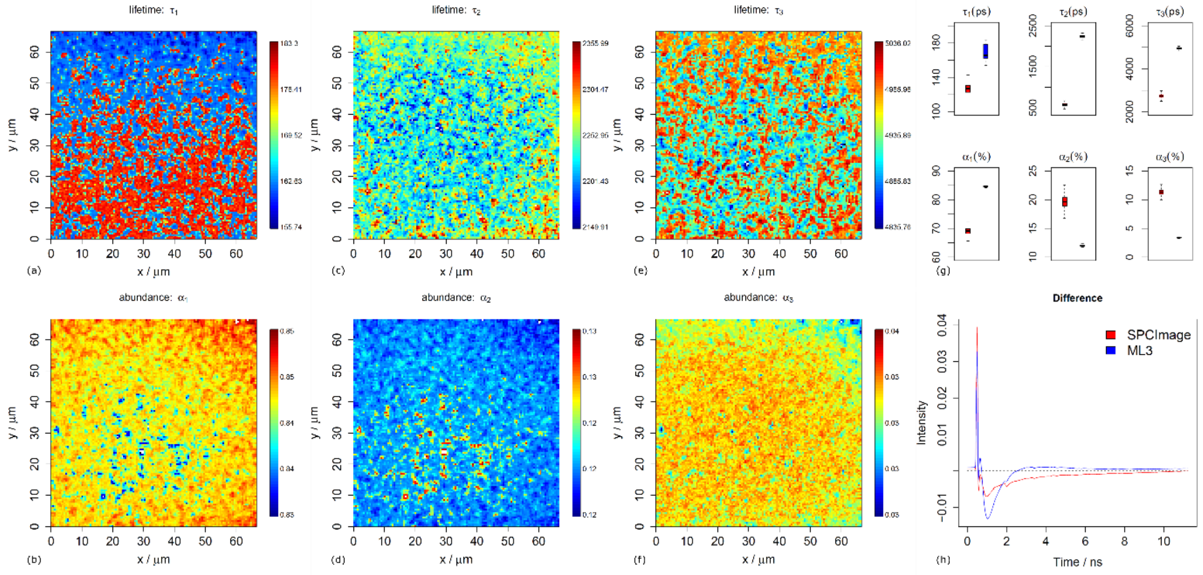

Figure S12. Results of the 3-component analysis on FCS data. To ensure a good contrast, all false-color plots were generated based on 0.001~0.999 percentiles of the values to be visualized. (a-f) Lifetime and abundance of the three components. (g) Results of the ML method (blue) along with the results of SPCImage (red). (h) Difference between the means of the reconstruction and the raw data.

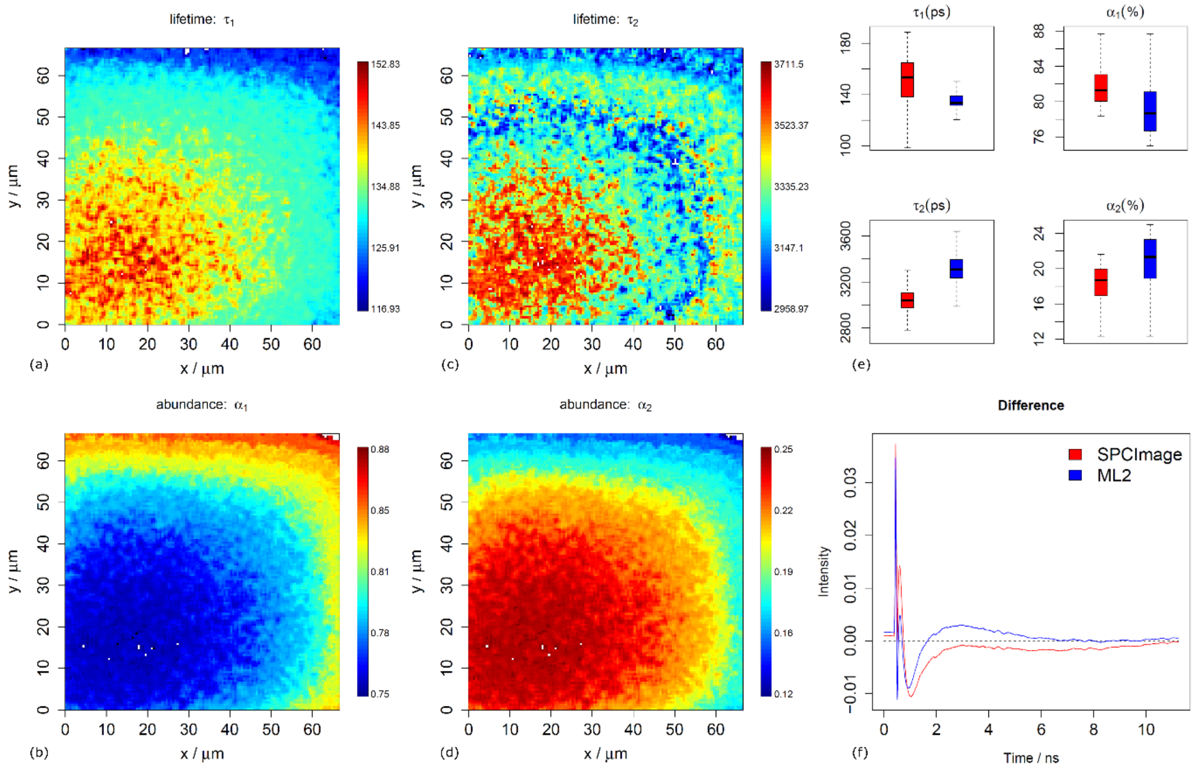

Figure S13. Results of the 2-component analysis on plasma data. To ensure a good contrast, all false-color plots were generated based on 0.001~0.999 percentiles of the values to be visualized. (a-d) Lifetime and abundance of the two components. (e) Results of the ML method (blue) along with the results of SPCImage (red). (f) Difference between the means of the reconstruction and the raw data.

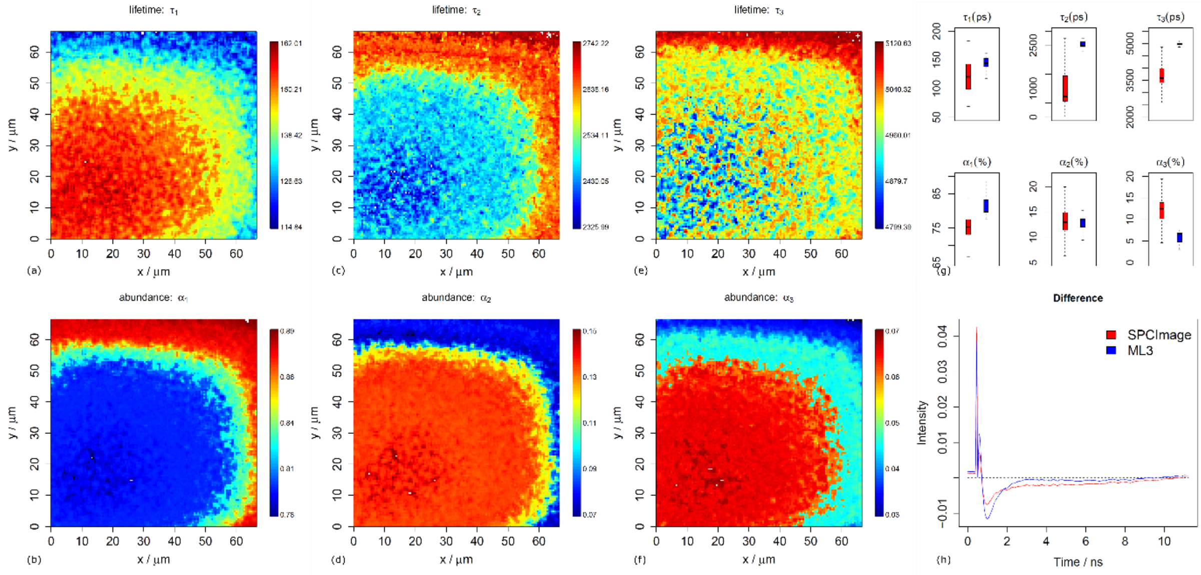

Figure S14. Results of the 3-component analysis on plasma data. To ensure a good contrast, all false-color plots were generated based on 0.001~0.999 percentiles of the values to be visualized. (a-f) Lifetime and abundance of the three components. (g) Results of the ML method (blue) along with the results of SPCImage (red). (h) Difference between the means of the reconstruction and the raw data.

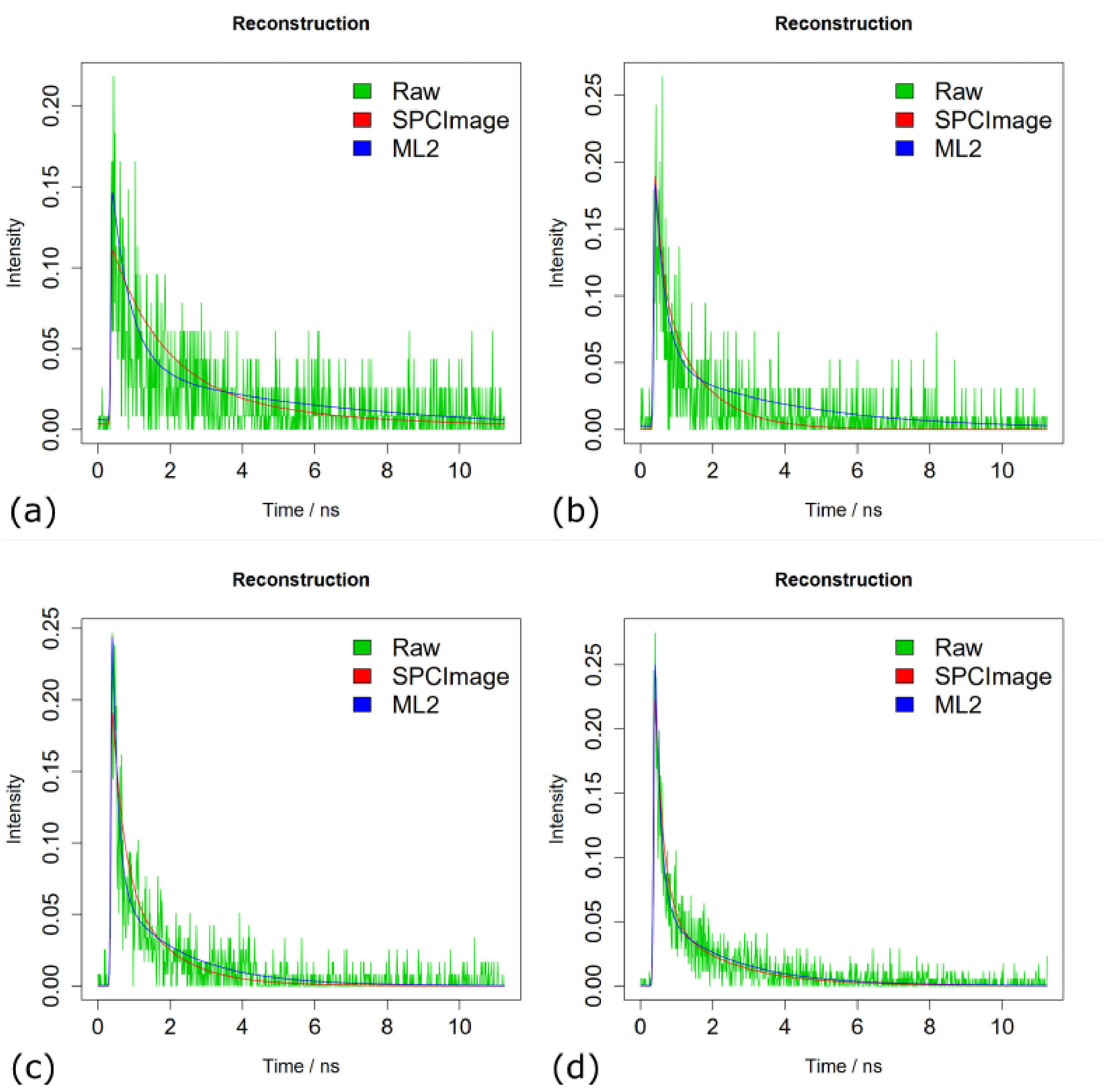

Figure S15. Example decays of different noise levels. In each subplot, the line series ‘Raw’ shows the decay after binning. The other two curves give the reconstructions according to 2-component analysis by SPCImage and the machine learning method, respectively.
